# Transport Response is a filial-specific behavioral response to maternal carrying in C57BL/6 mice

**DOI:** 10.1186/1742-9994-10-50

**Published:** 2013-08-14

**Authors:** Sachine Yoshida, Gianluca Esposito, Ryuko Ohnishi, Yousuke Tsuneoka, Shota Okabe, Takefumi Kikusui, Tadafumi Kato, Kumi O Kuroda

**Affiliations:** 1 Unit for Affiliative Social Behavior, RIKEN Brain Science Institute, 2-1 Hirosawa, Wako-shi, Saitama 351-0198, Japan; 2 Laboratory of Neuroendocrinology, Faculty of Medicine, University of Tsukuba, 1-1-1 Tennodai, Tsukuba, Ibaraki 305-8574, Japan; 3 Companion Animal Research, School of Veterinary Medicine, Azabu University, 1-17-71 Fuchinobe Chuo Ward, Sagamihara, Kanagawa 252-5201, Japan; 4 Laboratory for Molecular Dynamics of Mental Disorders, RIKEN Brain Science Institute, 2-1 Hirosawa, Wako-shi, Saitama 351-0198, Japan

**Keywords:** Mouse pup, Transport response, Calming response, Filial behavior, Maternal carrying, Mother-infant relationship, Parental behavior

## Abstract

**Background:**

A mother carries her young in many altricial mammals, such as cats, lions, rats and mice. During maternal carrying, the transported young assume a compact posture. We have recently shown that, in both humans and mice, the carried infants immediately calmed down and showed reductions in heart rate, distress vocalizations, and voluntary movement. The loss of the calming response in mouse pups hindered maternal retrieval efficacy. These findings suggested that the infant calming response functioned to reduce the maternal burden of carrying and was therefore conserved in a variety of mammalian species. However, it remains unclear how and when each component of this calming response develops and whether it is a filial-specific behavior.

**Results:**

We dissected various components of the carrying-induced responses in mouse pups, collectively called the “Transport Response” herein. We showed that during the second postnatal week, pups exhibited characteristic compact posture with limb ventroflexion. The body trunk remained paradoxically pliable, suggesting complex neural regulation throughout the body. Pups also showed an increased pain tolerance to a tail pinch during the Transport Response. Analyses of the developmental courses of distinct components of the Transport Response revealed the independent regulation of each component: in the first postnatal week, the cessation of ultrasonic vocalizations was exhibited prominently; in the second postnatal week, immobilization reached its peak; and toward the third postnatal week, the postural component became fully matured. At the end of the third postnatal week, when the pups are able to transport by themselves, the pups no longer exhibited the Transport Response.

**Conclusions:**

This study has revealed the mouse Transport Response as a complex set of behavioral and physiological components, each of which has a specific postnatal time window but is orchestrated in a well-matched manner with the maturation of ambulatory ability in the pups. These findings collectively indicate that the Transport Response is a filial-specific, innate behavioral reaction and is distinct from a simple reflex or defensive freezing response. The Transport Response could be a novel index of primitive filial attachment behaviors, acting to smooth mother-infant interaction.

## Background

Mammalian young are born immature and require intense parental care to grow up. Maternal behavior involves the provision of milk, body cleaning, thermoregulation, and protection from environmental hazards. In altricial species, newborns have limited ambulatory ability, and mothers often carry their young toward the nest or away from danger. For example, lionesses carry their cubs by picking them up by their necks with their teeth and transporting them for more than a few kilometers for nest relocation [1]. At the same time, it has been observed that the cubs become passive when they are held in this fashion and hang loosely with their hindlegs drawn up. Such a behavioral change in the young upon maternal transport has been noted in field studies of feral rodents [2,3] and in the primate Galago [4]. These studies also pointed to an artificial induction of this limp posture through manual carrying of the young that mimicked the maternal oral grasp. In laboratory rats, Brewster and Leon referred to this posture as the “transport response” [5]. Recently, we have examined the response of the young to maternal carrying in humans and in mice and shown that infants immediately show a reduction in crying, body movement and heart rate during carrying in both species [6]. Using pharmacologic and genetic interventions in mouse pups, we also investigated the upstream and downstream neural systems that regulate the calming response. Somatosensory and proprioceptive input signaling are required for induction, and parasympathetic and cerebellar functions mediate cardiac and motor output, respectively. The loss of the calming response hindered the maternal rescue of the pups, suggesting a functional significance for the identified calming response [6]. It was suggested that the calming response during maternal transport might increase the survival probability of the infant in cases of emergency escape by the mother-infant dyad and ultimately work to support the affiliative mother-infant relationship. However, it remains unclear how and when each component of this calming response emerges and whether it is a filial-specific behavioral response that is distinguishable from other immobility responses. To answer these questions, we further dissected the various behavioral and physiological components of the carrying-induced infant responses using C57BL/6 laboratory mice, collectively called the “Transport Response” herein. We use the term “Transport Response” with capitalization, in order to distinguish it from the previous definition of “transport response” based only on the specific compact posture [5]. In this study we use the extended term “Transport Response”, which includes all the pups’ responses to maternal-like carrying. As shown previously, the Transport Response could be induced in mouse pups by manually imitating maternal carrying [6], as observed in other rodent species [2,5]. This manual carrying method was preferable over maternal carrying for the standardized experimental observation of mouse pups and was therefore utilized in this study. In the last part of this study, we also used a semi-naturalistic behavioral task in which the mother carries the pups out of a cup.

## Results

### Components of the Transport Response: postural regulation

**Figure 1:**
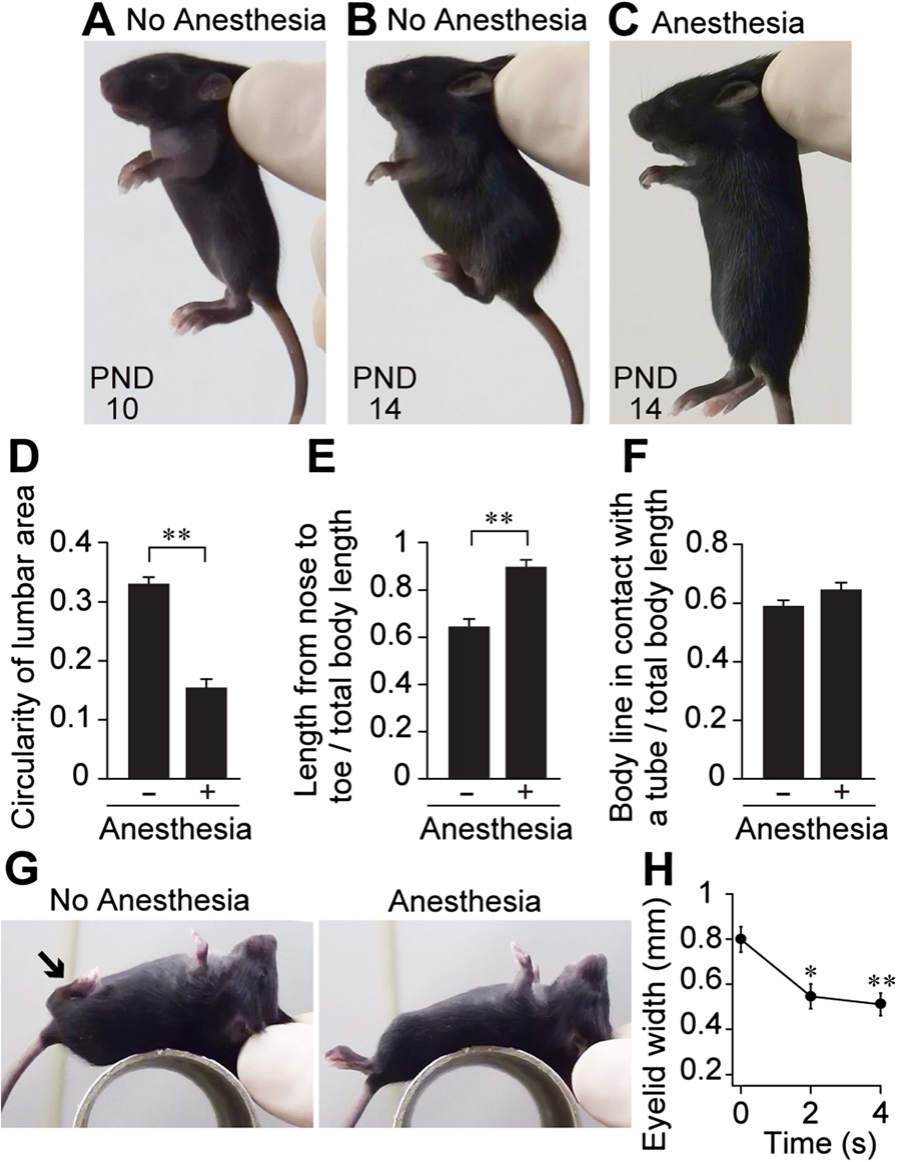
**Postural components of the Transport Response.** The typical posture during manual carrying of the undisturbed (No Anesthesia) pups at PND 10 **(A)** and PND 14 **(B)** compared with a PND 14 pup under general anesthesia with a 40 mg/kg pentobarbital injection **(C)**. **(D–F)** The mean ± SEM of the circularity of the lumber region **(D)**, the length from the nose to the toe **(E)** and the dorsal body length in contact with a tube normalized to the whole body length **(F, G)** during the immobilization period in the undisturbed and anesthetized pups at PND 14 are shown. *n* = 12 per group. **(H)** The mean ± SEM width between the upper and lower eyelids at 0, 2 and 4 s after the onset of immobility using PND 16 pups (*n* = 13). *p < 0.05, **p < 0.01.

Pups carried by an experimenter to mimic maternal oral carrying showed a characteristic compact posture, with flexion of the extremities as they grew (Figure 1A,B). While the hindlimbs of the postnatal day (PND) 10 pups were relaxed, those of a PND 14 pup carried by an experimenter were ventroflexed from the level of the pelvis (Figure 1B). To quantify the degree of body compaction and pliability during the Transport Response, we compared the posture during the Transport Response with the posture of the totally atonic condition during picking-up, by general anesthesia of the same age of pups. The lower back was significantly more curved in the normal PND 14 pups than that of the PND 14 anesthetized pups (*t*_(20.651)_ = 9.7963, *p* < 0.01, Figure 1D). Consequently, the entire body of a normal PND 14 pup maintained its compact position during the Transport Response, as measured by the nose to the toe length during the Transport Response compared with the nose-toe length under general anesthesia (*t*_(21.957)_ = –5.7821, *p* < 0.01, Figure 1E). The muscular tone required to maintain this compact posture was apparent when the posture of a normal PND 14 pup (Figure 1B) was compared with that of a pup of the same age under general anesthesia (Figure 1C).

When the carried pups were placed back on a tube in a supine position, the backs of the pups conformed to the curvature of the tube without resistance; this was similar to the behavior of the anesthetized pups (*t*_(21.784)_ = –1.9323, *p* = 0.066, Figure 1F, G; note that the hindlimbs of the left pup are maintained in a ventroflexed position in Figure 1G). These data indicated that despite the postural maintenance, the trunk was not all rigid during the Transport Response. In particular, the neck and the upper-body trunk were flexible and pliable. Moreover, the eyes of the PND 16 pups became progressively narrowed after manual carrying (Freidman test: Chi-square = 17.4286, df = 2, *p* < 0.01, Figure 1H). This data was consistent with the previous anecdotal observations that the transported pups often kept their eyes close (see pictures in [2,5]).

### Components of the Transport Response: apparent analgesia

**Figure 2:**
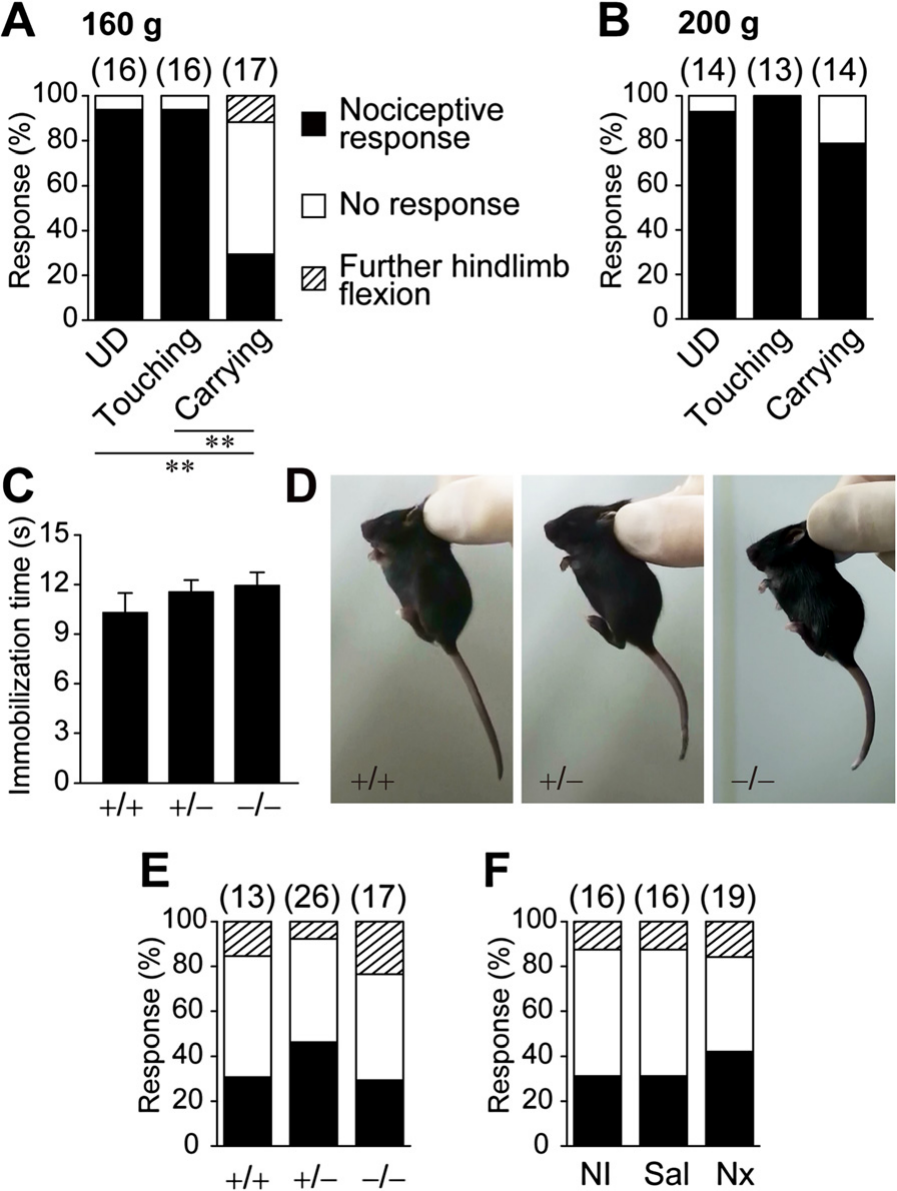
**Inhibition of the nociceptive response during the Transport Response. (A, B)** The pups’ responses to the noxious tail pinch at PND 13. The tail was pinched by an artery clip with 160 g **(A)** or 200 g **(B)** of force during the undisturbed condition (UD), after a 10-s immobilization between the experimenter’s fingers on the floor (Touching) or after a 10-s immobilization by manual carrying (Carrying). **(C–E)** The length of immobilization time during manual carrying **(C)**, the representative posture during manual carrying **(D)**, and the pups’ responses to the tail pinch **(E)** in wild-type (+/+), heterozygous (+/–), and homozygous (–/–) *Oprm* knockout mice at PND 13. **(F)** The responses of C57BL/6 pups to the tail pinch after a 10-s immobilization by manual carrying in pups that received no injection (NI), a saline (Sal) injection, or a naloxone (Nx) injection. The number of tested pups is provided in the parentheses. **p < 0.01.

Another possible feature of the Transport Response was the behavioral insensitivity to pain, as suggested through our daily handling of the mouse pups for procedures such as biopsy for DNA genotyping. To directly measure the pups’ apparent pain threshold, the tails of the pups were pinched by a clip with a known pinching force under three different conditions: manual carrying (Carrying), gentle touching between the experimenter’s fingers on a paper towel (Touching; see Manual touching in Additional file 1 as an actual maneuver) or under undisturbed conditions (UD; Figure 2A).

Pinching the tail using an artery clip with a 160 g pinching force elicited nociceptive responses in most of the pups in the UD and Touching groups. The pups responded to the pinch by squealing, rushing forward, or by turning back toward the tail and biting the clip (Figure 2A; Manual touching in Additional file 1). In contrast, only 5 of the 17 pups (29.4%) that were manually carried showed nociceptive responses. The other 10 pups (58.8%) exhibited no postural changes (Manual carrying in Additional file 2), and the remaining 2 pups (11.8%) further flexed their hind limbs (*p* < 0.01, Fisher’s exact test, Figure 2A).

This apparent analgesic state during the Transport Response demonstrated a ceiling effect, as the nociceptive response to the tail pinch with a clip of 200 g pinching force did not differ between the groups (*p* = 1 in UD vs. Touching, *p* = 0.074 in Touching vs. Carrying, *p* = 0.3 in UD vs. Carrying, Fisher’s exact test, *p-*value adjustment by Holm’s method, Figure 2B), suggesting an increase in the pain threshold.

To determine whether opioid signaling was involved in the apparent analgesia during the Transport Response, we first examined pups from the μ-opioid receptor knockout (*Oprm*^–/–^) mouse line for their responses to manual carrying and the tail clip. *Oprm*^–/–^ pups developed with no gross differences from the other genotypes in terms of appearance and weight gain (*F*_(2, 15.2)_ = 0.67, *p* = 0.52 at PND 10, *F*_(2, 17.15)_ = 1.88, *p* = 0.18 at PND 13). Mutant pups at PND 13 showed a normal Transport Response, including inhibition of voluntary movement (*F*_(2, 41.71)_ = 0.64, *p* = 0.53, Figure 2C) and compact postural adaptation (Figure 2D) when compared with their wild-type littermates. We carried pups of each genotype to induce the Transport Response and then pinched their tails with a clip of 160 g pinching force. There were no significant differences in nociceptive response types between the genotypes (No response: 47.06% of *Oprm*^–/–^, *p =* 0.31 in *Oprm*^+/+^ vs. *Oprm*^+/–^, *p =* 0.10 in *Oprm*^+/–^ vs. *Oprm*^–/–^, *p =* 0.89 in *Oprm*^+/+^ vs. *Oprm*^–/–^, Fisher’s exact test, *p-*value adjustment by Holm’s method, Figure 2E), suggesting that the apparent analgesic effect during the Transport Response persisted under the lack of the μ-opioid receptor. To further confirm the above finding, we also utilized the opioid receptor antagonist naloxone (Nx) [7]. The nociceptive responses to the tail pinch during the Transport Response were not significantly different between the C57BL/6 PND 13 pups injected with either Nx or saline (Sal) (*p =* 1 in no injection (NI) vs. Sal, *p =* 0.24 in Sal vs. Nx, *p =* 0.24 in NI vs. Nx, Fisher’s exact test, *p-*value adjustment by Holm’s method, Figure 2E). These results suggested that the expression of the nociceptive response is suppressed during the Transport Response via a non-opioidergic mechanism.

### Ontogeny of the calming response

**Figure 3:**
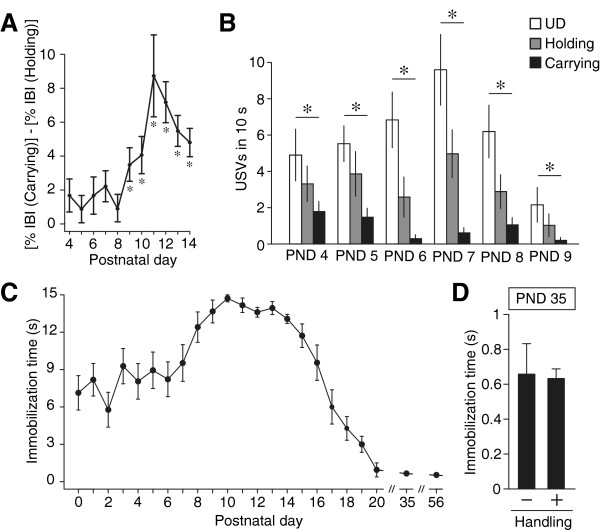
**Ontogeny of the IBI difference, USVs and immobilization during manual carrying. (A)** Ontogeny of the inter-beat interval (IBI) difference between Carrying and Holding during the first two postnatal weeks (*n* = 96). **(B)** Comparison of the number of ultrasonic vocalizations (USVs) emitted during the Undisturbed conditions (UD), Holding or Carrying from PND 4 to PND 9 (*n* = 36). **(C)** The mean ± SEM immobilization times during manual carrying (black filled circles, at PND 0–20, 35, 56; *n* ≥ 18 in each PND) are shown. **(D)** The mean ± SEM immobilization times of two groups of pups are shown. One group of pups was undisturbed except for a cage change once every other week (–). The other group of pups received continuous daily stroking by the experimenter’s hands for 20 s from PND 15 to PND 34 (+). All of the pups were picked up at PND 35 to examine their immobilization response (*n* = 18 for each condition). *p < 0.05.

To address whether the Transport Response is a filial-specific response, the ontogeny of the various components of the Transport Response were examined in detail using mouse pups. First, we compared the inter-beat interval (IBI in Figure 3A, the inverse of heart rate) during two different conditions using pups aged from PND 4 to PND 14. One condition was “Carrying”, during which the pups were gently held between the tips of the experimenter’s first two fingers and picked up in the air. The other condition was “Holding”, in which the pups were only held by the experimenter’s fingers. The amount of difference (%) in the inter-beat interval during the two conditions (Carrying minus Holding) showed no significant difference from PND 4 to PND 8 (*p* = 0.65, Figure 3A). From PND 9 onward, the inter-beat interval increased rapidly at the start of Carrying, and its difference between Carrying and Holding became evident (*F*_(5, 104)_ = 42.1, *p* < 0.001; Figure 3A). Next, we investigated the ontogeny of ultrasonic vocalization (USV) emissions during the Transport Response. The number of USV emissions was significantly lower in the Carrying condition from PND 4 to PND 9 compared with the Holding or UD (*F*_(5, 298)_ = 4.65, *p* < 0.001; Figure 3B).

We also examined the ontogeny of the immobilization response (Figure 3C). Of the total 547 mice, 271 male and 276 female pups between PND 0–20 were manually carried and held still in the air by the experimenter’s fingers for 15 s or until the pup started exhibiting anti-gravitational voluntary movements. At the end of the first postnatal week, cessation of the initial immobilization was almost always followed by struggling (rapid turning and shaking of the limbs and tail). Moreover, the struggling pups never returned to the immobilized state again. The mean time period of immobility was approximately 8–9 s during the first postnatal week. In the first few days after birth, the pups did not clearly inhibit their voluntary movement during manual carrying and would often start to move their extremities choppily. During the second postnatal week, the pups were immobile for longer periods of time. This immobilization effect peaked at PND 10, was gradually reduced after PND 15 and diminished by PND 20; the immobilization effect was not observed afterward (PND 35, 56 in Figure 3C). In C57BL/6 laboratory mice, this immobilization response could not be extended or evoked by continuous daily handling by the experimenter (*t*_(20.338)_ = 0.1365, *p* = 0.89, Figure 1D), although in adult rats such daily handling by experimenters could induce an immobilization response [5]. These data indicate that each component of the Transport Response, namely the cardiac deceleration, reduction of ultrasonic vocalization and immobility response, had a clear and separate time window within the preweaning period of mouse pups, and that did not observe in the adulthood.

### Ontogeny of postural regulation

**Figure 4:**
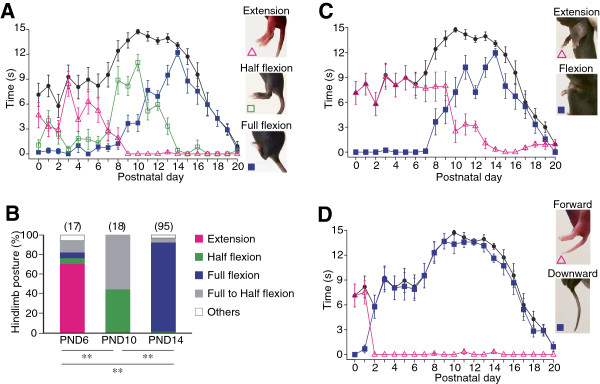
**Ontogeny of postural regulation during manual carrying.** The immobilization times (black filled circles in **(A)**, **(C)**, **(D)**) from PND 0 to PND 20 are the same as in Figure 3C and shown as a reference. **(A)** The mean ± SEM immobilization times that the pups maintained their specific hindlimb postures of extension (magenta triangles), half flexion (green squares) or full flexion (blue filled squares) are shown. **(B)** Comparison of the hindlimb postural types of PND 6, 10 and 14 pups. The postural types are categorized into five types: extension (magenta), half flexion (green), full flexion (blue), full to half flexion (gray) and others (white). The number of tested pups is provided in the parentheses. **p < 0.01. **(C)** The mean ± SEM immobilization times with an extended forelimb posture (magenta triangles) or a flexed posture (blue filled squares) are shown. **(D)** The mean ± SEM immobilization times with a forward (magenta triangles) or downward (blue filled squares) directed tail posture are shown. *n* ≥ 18 in each PND in **(A)**, **(C)**, **(D)**.

Next, we examined the ontogeny of the characteristic postural regulation described above, focusing on the hindlimb, forelimb and tail. Most of the pups immobilized with a symmetrical hindlimb posture; an asymmetrical posture was observed in only 8% of the 517 pups (data not shown). The symmetrical hindlimb postures were classified into three categories (Figure 4A): extension (magenta triangles), half flexion (green squares), and full flexion (blue filled squares). During the first postnatal week, most of the pups maintained the extended hindlimb posture. During PND 8–13, the pups would halfway flex their hindlimbs; alternatively, they would first fully flex their hindlimbs but then gradually let them down and extend them during the immobilization period. From PND 14 onward, most of the pups maintained their hindlimbs in the fully flexed position during the immobilization period. These observations suggested that the postnatal period could be roughly subdivided into three groups according to the hindlimb posture, the first postnatal week, PND 8–13 and PND 14 onward. To confirm this, we compared the hindlimb postural types at PND 6, 10 and 14 (Figure 4B). There were significant differences in the composition ration of postural types among the three PNDs (*p* < 0.001, Fisher’s exact test, *p-*value adjustment by Holm’s method, Figure 4B). The major type of the hindlimb posture was the extended posture at PND 6, the half flextion at PND 10 and the full flexion at PND 14.

The forelimb posture during immobilization was analyzed by classifying it into three categories (Figure 4C): symmetric extension, symmetric flexion and rare asymmetric positioning (data not shown). The developmental course of forelimb flexion was essentially similar to that of hindlimb flexion; the pups extended their forelimbs until PND 7 and then gradually maintained their forelimbs fully flexed throughout the immobilization period.

The tail posture was classified into two positions during postnatal development (Figure 4D). First, the tail was extended forward at PND 0 and PND 1. Next, the tail was extended downward from PND 2 onward.

### Maternal retrieval and the concomitant pups’ responses

**Figure 5:**
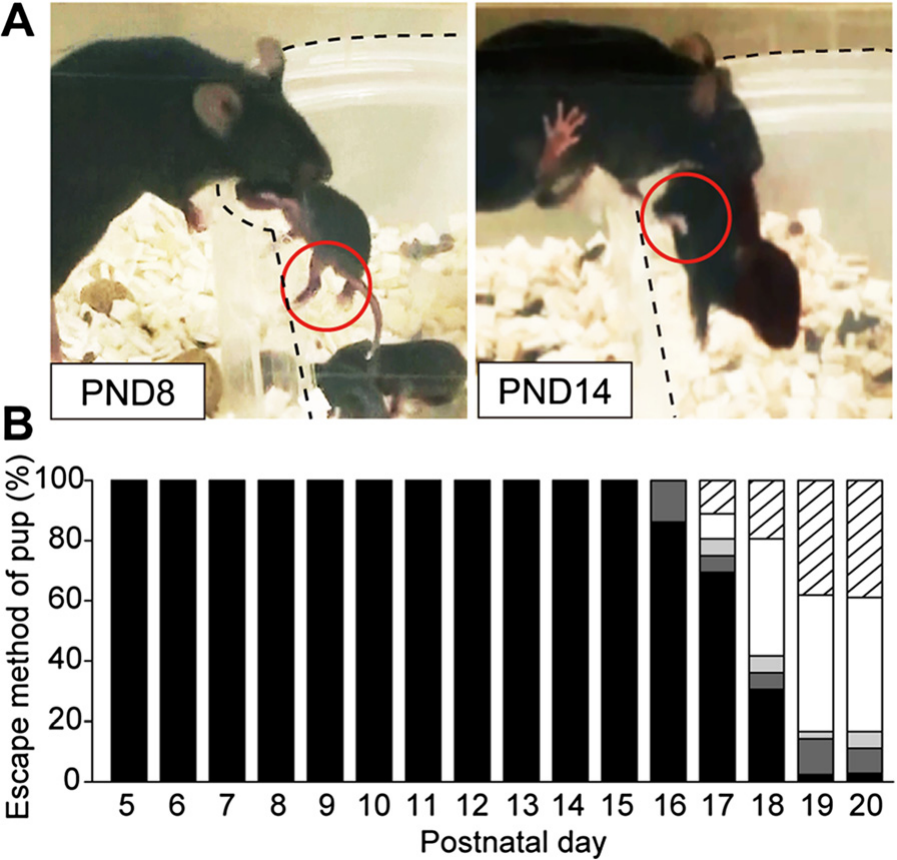
**The maternal rescue of pups from a cup and the pups’ responses. (A)** The pups’ postural responses during the task. The transparent plastic cup is outlined by dashed lines. The hindlimb was extended at PND 8 and flexed at PND 14 (red circles). **(B)** A proportion of the pups escaped from the cup in various manners: maternal rescue (black), both contributed (pup climbed up on the edge of the cup and the mother pulled the pup out; dark grey), the pup escaped by itself with a maternal attempt to grasp the pup (light grey), the pup escaped by itself with the mother in close attendance (white), the pup escaped by itself while the mother was away (stripe). *n* ≥ 24 for each age.

To examine the developmental course of mother-pup interactions in a more naturalistic setting, we used an experimental setup designated as the “maternal rescue of pups in a cup” [6] (Figure 5A). All of the pups were successfully rescued by their mothers until the pups were PND 16 (Figure 5B). As the pups grew older, they maintained their limbs in a more flexed position, as shown in Figure 4 and the right panel of Figure 5A. From PND 17 onward, the pups were able to climb up and get out of the cup independently (Figure 5B). The maternal attempts to orally retrieve the pup, support the pup’s escape by pulling the pup over the edge of the cup from the outside, or stay attentively near the pup until the pup got out remained until PND 19 or PND 20. This was the time period when almost all of the pups could independently get out of the cup within a few minutes. In this assay, no pup was left inside of the cup for more than 9 min after the start of the test session. These observations suggested that in this experimental setup, the development of the pups’ motor ability was a major determinant in diminishing the maternal retrieval rate. As shown in Figure 4, the attenuation of the immobilization response progressed in parallel with the pups’ increased motor ability. The expression of the Transport Response in the mouse pups coincided well with the pup’s development when they needed maternal rescue for their transport.

## Discussion

### Components of the Transport Response and its ontogeny

Our results have roughly subdivided the postnatal development of the mouse Transport Response into four phases.

The first phase approximately corresponds to the first postnatal week, when the pups do not show a clear immobilization and body compaction, but do calm down by a reduction in USV emissions during manual carrying. The pups during the first postnatal week are small and light, so that the mother may carry them easily even if the pups are moving during carrying.

The second phase corresponds to the second postnatal week and is characterized by passive adaptation, with heart rate reduction, robust immobilization and a relative insensitivity to the environment. It should be noted that the Transport Response is elicited by the force of grasping the neck, which is just sufficient to lift up the pup. Considering the limited cognitive and physical abilities of mouse pups by the second postnatal week, pups may show the Transport Response whenever they are lifted in the air without feeling much pain. This may be because they are most likely moved by their caretaker in this way; regardless, they do not have much chance to escape from the carrier by struggling, even when the carrier was a predator. In our previous study, disturbance of immobilization hindered the efficacy of maternal retrieval [6]. The pliability of the trunk may have also contributed to the ease of maternal carrying, especially when the mother had to drag the pup over bumps or through a narrow tunnel in a natural environment.

In the third phase, which lasts from the end of the second postnatal week to the first few days of the third week, each pup grow to approximately one-fourth of the maternal body weight (mean body weight: 6.6 g at PND 13). Passive immobilization along with active postural regulations, including limb ventroflexion and body compaction, may be required for the mother to carry her pup efficiently. In addition, apparent analgesia was observed during the Transport Response. A possible function of the apparent analgesia could be hypothesized as not to intervene the maternal carrying in states of emergency, such as during nest destruction [8]. In such an emergency escape, remaining calm by suppressing even nociceptive responses could help mother to relocate pups quickly and ultimately be beneficial for the pup’s own survival. Future evaluation will be required to test this hypothesis. This apparent analgesic effect remained in *Oprm*^–/–^ pups and naloxone-injected pups, suggesting regulation through a non-opioidergic mechanism. In connection with this, rat pups display apparent analgesia during suckling via a non-opioidergic mechanism [9]. A previous study with human infants revealed that having skin-to-skin contact with their mothers was a potent intervention against the pain experienced during a heel stick [10]. Increased pain tolerance observed in these various mother-infant interactions may share some common neural mechanism(s).

During the last phase, which corresponds to the remainder of the third postnatal week, the eyelids of the C57BL/6 mice were fully opened and the pups’ mobility became more mature, so that they were able to visually orient and travel by themselves. The pups’ immobilization response declined and reached the adult level by the time of weaning; accordingly, the mother refrained from orally retrieving its pups. These findings showed that mouse Transport Response is a composite of calming responses unique to preweaning pups. Our data support the notion that the infant Transport Response functions to facilitate maternal carrying as a filial contribution and to ultimately increase the probability of the pups’ own survival.

### The extended term “Transport Response” used in this study in comparison to previous studies

For the name of the described phenomenon or the pup’s responses to transport collectively, we used the term “Transport Response” out of respect for the pioneering study conducted by Brewster and Leon [5]. However, we want to extend the definition from Brewster’s study. Brewster and Leon referred only to the postural changes during manual mimicking of the mother’s grasp as the “transport response” [5]. In contrast, in the present study, we propose the term “Transport Response” as a collection of *all* of the responses evoked by transport in the transported young, including trunk curvature and pliability, limb flexion, immobilization, and other physiological changes. Care should be taken not to confuse this extended definition of the “Transport Response” from other contexts in previous literature as described below.

To the best of our knowledge, the oldest designation of the infant response to maternal carrying was made in Eible-Eibesfeldt’s field study of desert mice (*Meriones persicus*) [2]. To describe the response of the young to maternal transport, he used the German term “Tragstarre” [2], which literally means “carry–rigidity.” We hesitated to use this term, not only because it was not in English, but also because it included the term “rigidity” and was misleading for the limp and relaxed posture of the transported young. Previously, Brewster and Leon wrote: “When a Norway rat mother picked up a pup by its dorsal skin to transport it to a new nest site, the pup adopted a characteristic posture while being carried. This posture, referred to below as the “transport response” involves the extension and adduction of both forelegs against the body and the flexion of both hindlegs and the tail to the body” [5]. Moreover, their experimental coding of the transport response was performed “by allowing 5 possible points for the response: 1 point for each foreleg extended and adducted, 1 for each hindlimb flexed against the body, and 1 for the tail curled between the legs and toward the belly”. Therefore, the term “transport response” (Brewster & Leon, [5]) means only the postural regulation but did not include immobility or other aspects of the pup’s responses to transport. However, though without quantification, Brewster and Leon noted the immobility response during manual carrying in their text. Accordingly, we believe that our “Transport Response” in mouse pups is orthologous to Brewster and Leon’s “transport response” in rat pups.

More recently, the same term, “transport response (*TR*)” has been used in a series of studies performed by Wilson and colleagues in rats [11]. These studies consistently used the same coding method as the original study by Brewster and Leon. However, the induction maneuver utilized by Wilson was different from the one used in our study or from actual maternal transport, and varied even among their own studies: “all rats first were tested for *TR* intensity. This consisted of an experimenter grasping the pup by the nape of the neck between the experimenter’s thumb and first two fingers and firmly squeezing [12]”; and, “subject was grasped firmly by the nape of the neck and waved back and forth laterally to induce a *TR* of 4 or greater for a total time of 2 min (*TR* group) [13]”. Therefore, we assumed that the *TR* observed by Wilson and colleagues might not always be the same biological entity as the “Transport Response” described in our study.

### Distinction from other types of immobility

The response of the young to maternal transport has also been occasionally referred to as a type of behavioral arrest or immobility response [14,15], which includes tonic immobility (including freezing by fear and “animal hypnosis”) [16,17], dorsal immobility [18], and clamp- [19] or bandaging-induced immobility [14]. In particular, the limb posture regulation during the Transport Response and the dorsal immobility are similar, thus the clear distinction between them has not been made in the previous literature. Here we propose that the infant Transport Response to maternal carrying is distinguishable from other types of immobility for five main reasons: 1) the Transport Response is limited to the postnatal period. In contrast, tonic immobility is developed after the third postnatal week in deermice [20] and rabbits [21]; 2) the way of holding the subject animals required for induction is different between the Transport Response and other immobilization types. To induce the Transport Response, the pups are gently picked up just enough to lift the pup’s body up into the air. In contrast, tonic immobility is induced by inverting the animals and pressing them down [16,22]; dorsal immobility is induced when the animals are firmly grasped [23] or by tightly bandaging around the neck [14]; 3) the Transport Response does not require the vestibular input as shown in our previous study of labyrinthectomized mouse pups [6]. On the other hand, vestibular stimulation is necessary for dorsal immobilization in adult rats [24]; and 4) the remarkable pliability of the trunk during the Transport Response are the opposite of the motor rigidity in the dorsal and tonic immobility [15,18]; 5) the eyelids are rather closed in our study as well as in the previous anecdotal observations in the Transport Response, while the eyelids are widely opened in the dorsal ([18,24]) and the clamp-induced immobility ([25,26]). These data consistently suggested that the Transport Response is a distinct type of immobilization from dorsal or tonic immobility. These characteristics of the pups’ Transport Response can be explained by the relaxed and affiliative nature of the mother-infant interaction, which is in contrast to the extreme stress or predation that induces the defensive types of immobility. This notion, however, does not preclude the possibility that the motor output of the Transport Response may share the common neural pathway with the other immobility responses, in particular the dorsal immobility.

### The Transport Response as a novel innate behavioral index of a pup’s development

Although rats and mice are widely used experimental animals, the understanding of the behavior and physiology of developing pups is still limited compared to that of adult animals. Because pups have immature motor and cognitive abilities, there have only been a few behavioral testing paradigms available at specific developmental stages. Here, we identified multiple components of the mouse Transport Response, each of which is testable at a clear postnatal time window. In addition, we established two types of simple experimental methods that are compatible with specific-pathogen-free animal breeding conditions: first, the manual carrying task (in which the experimenter’s fingers mimic maternal oral grasping) can be used to screen for the abnormal sensory, motor, autonomic and central nervous system development relevant for the Transport Response; and second, the maternal rescue task (semi-naturalistic behavioral task in which the mother carries the pups out of a cup) can assess the pup’s response to maternal carrying as well as maternal performance in the actual mother-infant interaction. The maternal rescue task would be very useful for evaluating the relevant phenotypes of genetic mutant mouse lines in a normal breeding cage.

## Conclusions

This study systematically defined and quantified the Transport Response of mouse pups during postnatal development for both the active (the postural maintenance with flexed limbs) and the passive (calming responses and pliability) components of transport. The developmental course of each component of the mouse Transport Response has a distinct time window that reasonably corresponds with the physical maturation of the pup and concomitant changes in the maternal retrieval behavior. Our data indicate that the Transport Response is a filial-specific innate response. Additionally, a unique set of sophisticated motor controls placed throughout the body accompanied the apparent analgesia, which cannot be explained as a spinal reflex or a simple freezing behavior. The Transport Response can be an important component of primitive filial attachment behaviors, acting to smooth mother-infant bonding.

## Methods

### Experimental overview

Additional file 3 is an overview of experimental design to understand the mouse Transport Response.

### Animals

All animal experiments were approved by the RIKEN animal experiment committee. C57BL/6 mice were purchased from Japan SLC and CLEA Japan. Genetic mutant pups named B6.129S2-*Oprm1*^*tm1Kff*^/J were obtained from the Jackson Laboratory. Mice were maintained under a 12-h light/dark cycle (lights-on 08:00) with food and water *ad libitum*. C57BL/6 pups were culled to six containing males and females during PND 1 and PND 4 except for Figures 2C–E. Each genetic mutant pup received an injection of animal tattoo ink (Natsume, Japan) into the forepaw or the footpad to distinguish them from one another, and a small piece of the tail was collected for genotyping PCR at PND 3 or PND 4. The number of mutant pups was culled to six at PND 5. PCR was performed twice to verify the results, once before culling and once after the experiments. Each pup was used only in a single experiment and was not reused for multiple experiments. In Figure 3D, pups were gently stroked by an experimenter from PND 15 to PND 34. All experiments were performed between 10:00 and 12:00.

### Measurement of postural components during manual carrying

In this task we specifically tried to imitate maternal carrying in terms of holding strength; the dorsal skin of the pup’s neck was pinched with just enough force to pick up the pup but not too much, so that no mark was made on the skin after release [6]. We used powder-free latex gloves (Diamond Grip, Microflex, Reno, Nevada) for the manual carrying for animal safety regulation of RIKEN and to increase the friction at the finger tips. Postural coding and measurement of its maintenance time were performed for each limb and tail using still images that were extracted from a movie taken during the pups’ immobilization. These images were then processed using ImageJ software (NIH).

Anesthetized pups were prepared by sodium pentobarbital injection (40 mg/kg) (Kyoritsu Seiyaku, Japan). Measurement methods of the circularity of the lumber region, the body length and the width of eyelid were displayed graphically in Additional file 4. The circularity of the lumber region was determined using two index lines (lines 1 and 2 in Additional file 4A). Line 1 connected the caudal edge of the eyelid and the base of the tail. Line 2 was perpendicular to the middle point of line 1. The circularity of the lumber region was measured using the border line separated line 1 and line 2 (a red double-headed arrow in Additional file 4A). The body length during immobilization was determined through two processes. First, the length between the top of nose and the color border of the smallest toe was measured (a blue double-headed arrow in Additional file 4A). Next, the length was normalized to its own length from the nose to the anus under an anesthetized condition. The length from the nose to the toe and the length of the dorsal trunk in contact with the tube (which was an index of trunk pliability) were measured and then normalized by the length from the tip of the nose to the anus. The widths of the eyelids of each pup during manual carrying were measured using still images that were extracted from the movie file at 0, 2 and 4 s after the onset of immobilization. Because the eyelid was not fully open until the end of the second postnatal week in the C57BL/6 mouse pup, the measurement of eyelid width was performed using the PND 16 pups to obtain a clear observation. Line 3 (Additional file 4B) connected the rostral and caudal edges of the eye. Subsequently, a new line was drawn perpendicular to the middle point of line 3. The width of the eyelid was measured between the two intersecting points of the drawn line and eyelid (a yellow double-headed arrow in Additional file 4B).

### Analysis of the pain response during manual carrying

The handgrips of the artery clips (Natsume) were polished to modify the clipping power as described [27]. Each pup was assigned to one of the following three conditions: 1) the pup was undisturbed in its home cage (UD); 2) the pup was placed on a paper towel and its lateral body walls were gently held between the experimenter’s fingers (Touching) using the behavioral characteristics of pups that make them huddle with the warmer target [28]; or 3) the pup was picked up to induce the Transport Response (Carrying). The tails of the UD pups were pinched immediately following their removal from their home cages. The tails of the Touching and Carrying pups were pinched with a 160 g or 200 g clip for up to 5 s after a 10-s immobilization period was confirmed. To examine the involvement of signal cascade via μ-opioid receptor, *Oprm*^+/+^, *Oprm*^+/–^ and *Oprm*^–/–^ pups were used. The Transport Response was induced by manual carrying and the immobilization time was counted using the same method as described in Ontogeny of immobilization and postural regulation during the Transport Response in this Methods section. Each pup received a tail pinch with an artery clip of 160 g pinching force for up to 5 s after a 10-s immobilization period. The pinching force used was determined by our preparatory experiments. In the experiment using the opioid antagonist, C57BL/6 pups received 5 mg/kg naloxone hydrochloride (Sigma, St. Louis, MO) intraperitoneally 10 minutes before the tail pinch test.

### Measurements of cardiac function using the manual carrying method

For a general description of the measurements of heart rate and USVs using the manual carrying method, please refer to Esposito et al [6]. Each pup was handled by the experimenter under three different conditions: (1) the pup was undisturbed (UD), (2) the pup was grasped (as during Carrying) but was not lifted (Holding), (3) the pup was grasped with two fingers by the nape of the neck and lifted (Carrying). Two custom-made electrodes (0.7 mm ϕ, Unique Medical, Japan) were placed on the proximal part of both forelimbs and connected with an Electrocardiogram (EKG) data collecting system (ATC-402 and UAS-308S, Unique Medical). Each pup was then handled by the experimenter in the series of above-described three different conditions, which were randomly presented four times each for 20 s. All EKG files were analyzed using the Unique Acquisition (Unique Medical CO, LTD) software to identify and label each QRS complex. All data were reviewed by one analyst and edited. Artifacts and ectopic complexes were deleted. Subsequently, the duration of each inter-beat interval (IBI) was extracted. The IBI represents the time elapsed between two successive heart beats; it is an inverse of the heart rate. In this study, we report the percentage (%) of the amount of change in the IBI from Carrying to Holding.

### Ultrasonic vocalizations

Each pup was placed in a metal receptacle (4 × 19 × 25 cm) that contained cage bedding (α-dri) in an acoustically insulated room. After 20 s (initial baseline), the pups were handled by the experimenter’s fingers in the three different conditions (UD, Holding and Carrying*)*. Each of the three conditions was presented twice for 10 s and was interspersed with a 20-s interval of non-stimulation (baseline). These 20-s intervals were inserted to control: (1) in general, any effect of a stimulation (e.g., Carrying) on the next stimulation (e.g., Holding); (2) in particular, the maternal potentiation effect ([29]: this was shown in rat pups and highlights how pups tend to emit more USVs right after they are grasped and transported). In our preliminary study, the maternal potentiation effect was confirmed to last for approximately 5 to 10 s. Ultrasonic sounds between 10 to 200 kHz were recorded and analyzed using a condenser microphone (UltraSoundGate CM16/CMPA, Avisoft Bioacoustics, Germany), an A/D converter (UltraSoundGate 116, Avisoft Bioacoustics) with a sampling rate of 300 kHz, and a sound analysis system (SAS Lab Pro, Avisoft Bioacoustics). A CCD video camera was used for monitoring, and the signal was recorded using the software Honestech VHS to DVD 2.0 SE (Honest Technology). A research assistant subsequently analyzed the videos, and the amount of USVs during the three different conditions (UD, Holding and Carrying) was calculated. USVs in the different conditions were normalized over a 10-s interval.

### Ontogeny of immobilization and postural regulation during the Transport Response

Each pup was picked up only once to examine the Transport Response from PND 0 to PND 20, 35, and 56 (n ≥ 24 in each PND). The mice were manually picked up for 15 s and were recorded with a Handycam HDR-SR12 video camera (Sony). The filming of the mice was performed at the same distance and angle from the camera and was recorded using a scale. The movie replay and editing were conducted using the Picture Motion Browser (Sony) and Premiere Pro CS4 (Adobe, CA) software programs.

The immobilization times were manually measured using stopwatches for the latency of the initial struggle response (i.e., the rapid and anti-gravitational movement throughout the body) from pick-up. If the pup did not initiate struggling for 15 s, the pup was then put down and its immobilization time was documented as 15 s. The younger pups would sometimes slowly move their extremities along the gravitational force; that is, they could not maintain their limb postures throughout the immobility time period and would let their limbs down from flexion to extension. Such movements were not regarded as a struggling response.

Postural types of the hindlimb, forelimb and tail were recorded in a pup that showed a 1-s or longer immobilization. The hindlimb postures were categorized into four types: extension, half flexion, full flexion and asymmetry. To compare the hindlimb postural types, PND 6, 10 and 14 pups that showed a 1-s or longer immobilization were categorized into five types: extension, half flexion, full flexion, gradual change from full to half flexion, and other atypical postures (such as asymmetry). The forelimb posture types were extension, flexion and asymmetry. The tail posture types were forward, downward and backward extensions. When the pup’s posture would change gradually during the immobility periods, the maintenance times of these two different postures were separately measured. Asymmetric postures of the hindlimb and forelimb and a backward/laterally extended tail were rarely observed (data not shown).

### Observation of maternal retrieval and the concomitant pups’ responses

The experiment performed in Figure 5 was described elsewhere [6]. In brief, to increase the visibility of the subjects, one side of a transparent plastic cup (125 mm ϕ × 85 mm) was transected (basal area 122.7 cm^2^, height 8.5 cm) and fitted to the cage wall. The video was recorded at an angle to view the open side of the cup. Next, to habituate the mother to the cup, the experimenter first put the mother in the plastic cup; the mother would quickly get out of the cup. This procedure was repeated several times during PND 4 to PND 6, before introducing the pups into the cup from PND 5 onward. In this way, the mother mice would be better habituated; all of the primiparous C57BL/6 mothers retrieved each pup within a few minutes from PND 5 onward. Every day from PND 5 to PND 20, three pups at a time were taken from their nests and placed into the cup to induce maternal retrieval; this was the first session. The other three pups were transferred to another cup that was set in a different cage of the same shape that was stuffed with the home-cage bedding. After the first session, the remaining three pups were introduced into the cup for the second session. These rescue sessions were video recorded using a Handycam HDR-SR12 (Sony, Japan), and the various parameters of the pups’ responses to the maternal retrieval behavior were measured by a frame-by-frame video analysis (25 frames per second) using the Free Video To JPG Converter (DVDVideoSoft, IL).

### Common procedures for video and statistical analyses

Video analyses were performed by at least two raters without any prior information about the experimental manipulations. Statistical analysis was performed using Welch’s t test, the Friedman test, and Welch’s ANOVA as appropriate, with significance set at *p* < 0.05 after *p*-value correction by Holm’s method. All statistical analyses were conducted using R 2.9.0 (R Development Core Team (http://www.R-project.org)).

## Abbreviations

Nx: Naloxone; NI: No injection; PND: Postnatal day; Sal: Saline; USV: Ultrasonic vocalization; UD: Undisturbed condition.

## Competing interests

The authors declare that they have no competing interests.

## Authors’ contributions

SY and GE designed the experiments and performed the study with the support of RO, YT and SO. KOK supervised the study with supports from TK (Azabu Uni) and TK (RIKEN). All authors read and approved the final manuscript.

## Supplementary Material

Additional file 1 A movie of an actual Touching maneuver and a pup’s response for the tail pinch during Touching. Click here for file

Additional file 2 A movie of an actual Carrying maneuver and a pup’s response for the tail pinch during Carrying. Click here for file

Additional file 3 An overview of experimental design to understand the mouse Transport Response. Click here for file

Additional file 4 A graphical explanation of posture measurement. Click here for file
