# Supplementary material for: Transport Response is a filial-specific behavioral response to maternal carrying in C57BL/6 mice
Source: Front Zool. 2013 Aug 14;10:50. doi: 10.1186/1742-9994-10-50 (PMC3751433; doi:10.1186/1742-9994-10-50)
Supplement: Additional file 3 — An overview of experimental design to understand the mouse Transport Response. [file 1742-9994-10-50-S3.docx]

Additional File 3

Overview of experimental design to understand the mouse Transport Response

| Analysis type | Componential analysis | | Ontogenic analysis | | |
| --- | --- | --- | --- | --- | --- |
| Study point  (Figure no.) | Postural component  (Figure 1) | Apparent analgesia  (Figure 2) | Heart rate, USVs and Immobilization change  (Figure 3) | Postural change  (Figure 4) | Change of pup’s response to maternal carrying  (Figure5) |
| Induction method | Manual carrying | | | | Maternal carrying |
