# Supplementary figures and images for: Transport Response is a filial-specific behavioral response to maternal carrying in C57BL/6 mice
Source: Front Zool. 2013 Aug 14;10:50. doi: 10.1186/1742-9994-10-50 (PMC3751433; doi:10.1186/1742-9994-10-50)

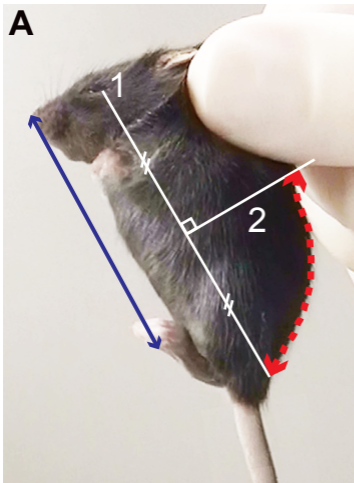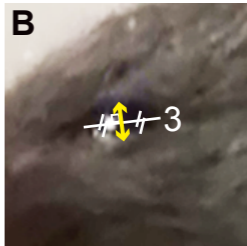

Supplement: Additional file 4 — A graphical explanation of posture measurement. [file 1742-9994-10-50-S4.pdf]
